# Liver-boosting potential: chicory compound-mediated silver nanoparticles for hepatoprotection—biochemical and histopathological insights

**DOI:** 10.3389/fphar.2024.1325359

**Published:** 2024-02-21

**Authors:** Ayesha Siddiqa, Rahmatullah Qureshi, Naveed Iqbal Raja, Imtiaz Ahmed Khan, Muhammad Zishan Ahmad, Shaista Rafique, Amir Ali, Ajaz Ahmad, Prashant Kaushik

**Affiliations:** ^1^ Department of Botany, Pir Mehr Ali Shah Arid Agriculture University, Rawalpindi, Pakistan; ^2^ Department of Veterinary Pathology, Pir Mehr Ali Shah Arid Agriculture University, Rawalpindi, Pakistan; ^3^ Department of Clinical Pharmacy, College of Pharmacy, King Saud University, Riyadh, Saudi Arabia; ^4^ Department of Vegetable Science, Chaudhary Charan Singh Haryana Agricultural University, Hisar, India

**Keywords:** silver nanoparticles, hepato-protective activity, liver diseases, chicory compound, histopathology

## Abstract

**Background:** Liver disease is a serious health concern in today’s world, posing a challenge to both healthcare providers and pharmaceutical companies. Most synthetic drugs and chemicals cause liver damage accounting for approximately 10% of acute hepatitis and 50% of acute liver failure.

**Purpose:** The present study aimed to evaluate the hepato-protective activity of an extract of chicory formulation assisted by silver nanoparticles against carbon tetra chloride (CCl_4_)-induced hepatic damage in rat’s liver.

**Methods:** Rats of the Wistar strain (Rattus norvegicus) were used to test the *in vivo* hepato-protective efficacy at various doses. Rats were randomly divided into nine groups, each containing six rats. The groups were as follows: first group (control), second group (CCl_4_), third group, silymarin (20 mg/kg of body weight), fourth group (CCl_4_+chicory) (1.75 mg/kg of b. wt), fifth group (CCl_4_ + chicory at the dose of 2.35 mg/kg), sixth group (CCl_4_ + chicory of 3.25 mg/kg), seventh group (CCl_4_ +AgNPs 1.75 mg/kg of b. wt.), eighth group (CCl_4_ + AgNPs 2.35 mg/kg of body weight), and ninth group (CCl_4_ + AgNPs 3.25 mg/kg of b. wt.). Blood samples were taken 24 h after the last administration (i.e., 30th day). The blood samples were analyzed for different serum enzymes such as ALP *(alkaline phosphatase)*, ALT (alanine transaminase), bilirubin (Blr), triglyceride, and cholesterol. Histology liver sections were performed.

**Results:** Treatment with AgNPs and chicory extract showed significant hepato-protective activity in a dose-dependent manner. In three doses, the chicory extract at a rate of 3.25 mg/kg of body weight significantly reduced elevated levels of biochemical markers in comparison to CCl_4_-intoxicated rats. Histology of the liver sections from CCl_4_-treated rats revealed inflammation of hepatocytes, necrosis, cytoplasmic degeneration, vacuolization, and a deformed central vein. The chicory formulation extract exhibited a remarkable recovery percentage in the liver architecture that was higher than the drug (i.e., silymarin). While treatment with AgNPs also repaired the degenerative changes and restored the normal form of the liver, chicory formulation extract possessed more hepato-protective potential as compared to AgNPs by regulating biochemical and histo-pathological parameters.

**Conclusion:** This study can be used as confirmation of the hepato-protective potential of chicory compounds for possible use in the development programs of drugs to treat liver diseases.

## Introduction

The liver is the largest glandular organ of the body whose functions are greater than any other organ (Sanjiv, 2002). It regulates a variety of physiological functions such as metabolism, bile secretion, and vitamin storage. In addition, this organ detoxifies various toxic substances and is involved in the synthesis of useful compounds (Latief et al., 2023). It is important in the maintenance of body functions in addition to the regulation of homeostasis, as this organ regulates various metabolic processes such as growth, disease resistance, nutrition delivery, energy flow, and reproduction. Being a detoxifying organ, it is vulnerable to constant attack by foreign molecules, resulting in liver failure (Kumar et al., 2021). Therefore, the maintenance of the liver’s health is important for the wellbeing of an individual. Liver disease is a serious health concern in today’s world, posing a challenge to both healthcare providers and pharmaceutical companies (Smuckler, 1975). Liver disorders are among the most serious health problems in the world (Malhotra and Singh, 2001). These can be classified as acute and chronic hepatitis (inflammatory liver disorders), hepatosis, and cirrhosis. Different toxic chemicals such as carbon tetrachloride, thioacetamide, certain chemotherapeutic agents, high alcohol consumption, and some microbes result in liver cell injury (Bharathi et al., 2023). CCl_4_ is a xenobiotic that is introduced into water as waste from different industries, causing hepatotoxicity in living creatures. It is frequently used to cause liver disease in various models to test hepato-protective medicines (Malhotra and Singh, 2001).

Synthetic medications available on the market for liver treatment cause many issues (Vairetti et al., 2021). Even the Food and Drug Administration (FDA) of the US has withdrawn or not approved drugs that cause liver ailments (Ostapowicz et al., 2002; Pandit et al., 2012). Over 1,000 drugs and chemicals have been found to be a cause of liver damage (Upadhyay et al., 2010; Porceddu et al., 2012), responsible for 10% of acute hepatitis and 50% of acute liver failure (Pandit et al., 2012). There is increasing concern about drug-induced hepatotoxicity, which also results in malnutrition, malfunctioning of organs, and mortality (Porceddu et al., 2012).

Liver treatment can be conducted with phytomedicines, as they are harmless, easily accessible, cost-effective, and minimally toxic with fewer side effects compared to conventional medicines (Agarwal et al., 2006; Nair and CHANDA, 2007; Doudach et al., 2022). Therefore, there is increasing interest in herbal medicines (Hussain et al., 2010). For thousands of years, medicinal plants have been used by local communities all over the world for the treatment of numerous ailments. Historically, Egypt, China, and Greece are the oldest civilizations with a rich history of herbal formulations.

Chicory (*Cichorium intybus* L.) is popularly used for liver disorders (Faraji et al., 2020). A poly-herbal compound is available in the market with the name Sharbat-e-Bazori (Khan et al., 2024), obtained from its seeds and roots, along with other herbs such as *Foeniculum vulgare* seeds and root, and seeds of *Cucumis melo* var*. utilissimus*, *C. sativa*, and *Tribulus terrestris* (Hayat et al., 2008). This compound is a famous Unani product that is used in various liver disorders such as hepatic cirrhosis, fat accumulation, hepatic steatosis, and end-stage liver disease (Faraji et al., 2020). It also helps with urinary tract infections and possesses lithotriptic, diuretic, and alkalinizing effects (Aziz et al., 2011).

Nanomedicine is an emerging field of science that is popularly used in various disciplines, including medicine, to provide cost-effective and novel therapies. Various researchers have used metal nanoparticles because of their broad application in medicine, biology, and physics (Caruthers et al., 2007). Among all metallic nanoparticles, silver nanoparticles (AgNPs) possess unique properties and are commonly used in experiments (Rai et al., 2009). AgNPs have antifungal, antibacterial, and antioxidant activities and are anti-inflammatory (Gurunathan et al., 2009; Wong et al., 2009). Silver nanoparticles have been studied for several therapeutic purposes, as they are less toxic and have increased biodegradability and bioavailability. Due to their small size, they can easily interact with biological systems.

However, scientific literature data supporting the folkloric use of the chicory formulation in liver diseases is not available, and its tentative mechanism(s) are still unknown. The present study therefore aimed to evaluate the potential *in vivo* hepatoprotective activity of aqueous extracts of chicory formulation against CCl_4_-induced hepatotoxicity.

## Materials and methods

### Preparation of chicory compound

The raw material used for the preparation of chicory formulation as defined in Unani Pharmacopoeia was obtained from a local market in Rawalpindi, Pakistan, identified in the taxonomy lab of the Department of Botany, Pir Mehr Ali Shah Arid Agriculture University Rawalpindi, Pakistan. To prepare the chicory compound, dead and decaying parts were removed from samples and washed in running tap water for the removal of dust. The plant parts (Table 1) were dried in the open air at room temperature and ground to a fine powder. Each ingredient of the chicory formulation was mixed in proportion, i.e., *C. intybus* (seed and root), *F. vulgare* (seed and root), and melon seeds (*C. melo*), according to Unani Pharmacopoeia (Shailajan et al., 2012).

**TABLE 1:** Ingredients of chicory formulation.

Plant species	Common name	Part	Wt. (g)
Cichorium Intybus	Seed	Kasni	8
Cichorium Intybus	Root	Kasni	8
Foeniculum Vulgare	Seed	Sonf	8
Foeniculum Vulgare	Root	Sonf	8
Cucumis melo	Seed	Tukhme kharboza	8
Cucumis sativus	Seed	Tukhm e khera	8

### Preparation of extracts

**FIGURE 1:**
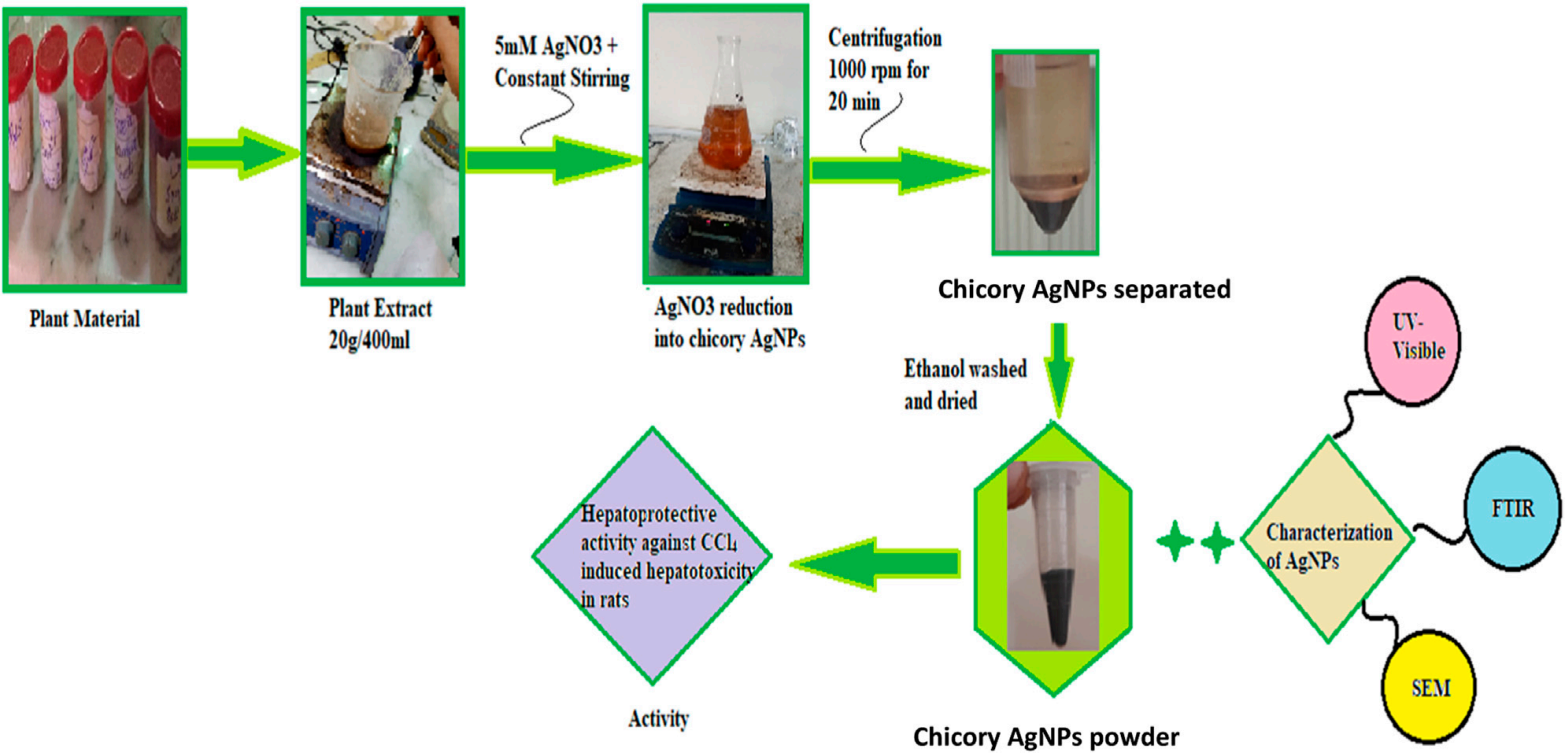
Process for synthesis of silver nanoparticles from chicory compound.

The plant extract was prepared with distilled water. For this purpose, 20 g of chicory formulation powder was added to 400 mL of distilled water in a separate flask. The flask was covered with foil paper and kept in a shaker for a day. The mixture was filtered using filter paper. After filtration, the dried extract was separated by the use of a needle and put in an Eppendorf tube. The residue powder was saved for future use (Figure 1).

### Synthesis of silver nanoparticles

For the synthesis of AgNPs, 0.34 g of AgNO_3_ was added to 1,000 mL of water to prepare a 5 mM solution of silver nitrate. Then 100 mL of plant extract was added to 900 mL of silver nitrate solution. The plant extract was added drop-wise with constant shaking in a conical flask. The synthesis of nanoparticles was carried out with a microwave oven to speed up the process of AgNP formation. The microwave was set at 600 W, and the solution was observed every 30 s until a color change appeared. The color change demonstrated the formation of silver nanoparticles. After this, the mixture was centrifuged at 10,000 rpm for 20 min to separate the impurities. After centrifugation, precipitated AgNPs were washed twice with ethanol to remove impurities. The pellet formed inside the centrifuge tube was collected carefully and dried (Figure 1) (Roy et al., 2015; Haryani et al., 2018).

### Characterization of silver nanoparticles

The formation of silver nanoparticles was marked by a change in color. A UV visible spectrophotometer (V-750 UV-Visible Spectrophotometer), Fourier transform infrared spectrophotometer (NICOLET 6700, Thermo, Waltham, MA, United States), energy dispersive X-Ray analysis (SIGMA model), and a scanning electron microscope (SEM, JSM5910 JEOL, Tokyo, Japan) were used for the characterization of synthesized silver nanoparticles.

### *In vivo* hepato-protective activity

Male albino rats of the Wistar strain (*Rattus norvegicus*), with weight of 175–250 g), were obtained from the animal house of the Department of Veterinary and Animal Sciences, Arid Agriculture University Rawalpindi, and used for the experimental study. Rats were kept in clean plastic cages in the laboratory on a standard pellet diet and tap water. They were allowed to adapt to the laboratory conditions for a week before treatment.

#### Preparation of doses and treatments

The solution of AgNPs was made in distilled water, and different doses of plant chicory formulation and AgNPs were administered to the animals. Silymarin (20 mg/kg of body weight) was administered as standard treatment (Nirala and Bhadauria, 2008).

#### Experimental procedure

After 1 week of acclimatization, rats were randomly divided into nine groups, each containing six rats. The detailed treatment layout is provided in Table 2. The first group was kept as normal rats (control), the second group was the CCl_4_-treated group (1 mg/kg of body weight), the third group, silymarin (20 mg/kg of body weight), the fourth group (CCl_4_ (1 mg/kg)+chicory (1.75 mg/kg of b. wt), the fifth group (CCl_4_ (1 mg/kg) + chicory at a dose of 2.35 mg/kg), the sixth group (CCl_4_ (1 mg/kg) + chicory of 3.25 mg/kg), the seventh group (CCl_4_ (1 mg/kg) +AgNPs 1.75 mg/kg of b. wt.), the eighth group (CCl_4_ (1 mg/kg) + AgNPs 2.35 mg/kg of body weight), and the ninth group (CCl_4_ (1 mg/kg) + AgNPs 3.25 mg/kg of b. wt.).

**TABLE 2:** Treatment plan for *in vivo* hepato-protective activity of AgNPs and chicory extract.

Treatments	Code
Control	T1
Negative Control (CCl4)	T2
Silymarin 20 mg/kg	T3
CCl4+ Chicory extract 1.75 mg/kg	T4
CCl4+ Chicory extract 2.35 mg/kg	T5
CCl4+ Chicory extract 3.25 mg/kg	T6
CCl4+ AgNPs 1.75 mg/kg	T7
CCl4+ AgNPs 2.35 mg/kg	T8
CCl4+ AgNPs 3.25 mg/kg	T9

All plant extracts were made in distilled water and administered through an oral route for four successive weeks. The amount of each extract was freshly prepared, weekly according to body weight changes, and plant extract was injected daily for 30 days. The CCl_4_-treated groups received oral administration of CCl_4_ (1 mL/kg per body weight) three times weekly for two consecutive weeks (Elshater et al., 2013).

The rats were anesthetized by using a cotton-wool ball soaked in diethyl ether and placed in a closed container. Rats were humanely sacrificed, and blood samples were collected to obtain the serum. Within half an hour of blood collection, the sera samples were obtained from the blood. The sera were kept in a deep freezer until analyzed.

### Blood biochemistry

The serum was used for the determination of markers of liver function tests (LFTs) such as alkaline phosphatase (ALP) and alanine transaminase (ALT) (Jalili et al., 2022). Cholesterol, triglyceride, and bilirubin were measured using diagnostic kits, and an auto-analyzer (Micro Lab 200, Merck) was used for the measurement.

### Histopathological examination

Small pieces of liver tissue in each group were collected in 10% neutral-buffered formalin. These tissues were processed and embedded in paraffin wax. Sections of 3–5 μm thickness were cut and stained with hematoxylin and eosin (H&E). The sections were examined microscopically for the evaluation of histopathological changes (Bancroft et al., 1994).

### Statistical analysis

The results were expressed as the mean ± S.D. The data were analyzed statistically by one-way ANOVA; *p* < 0.05 was considered significant in all cases. A post-hoc test was performed for multiple statistical comparisons between parametric data. The software Statistix 8.1 was used for the analysis of the data.

## Results

### Characterization of nanoparticles

#### UV-visible spectroscopy

**FIGURE 2:**
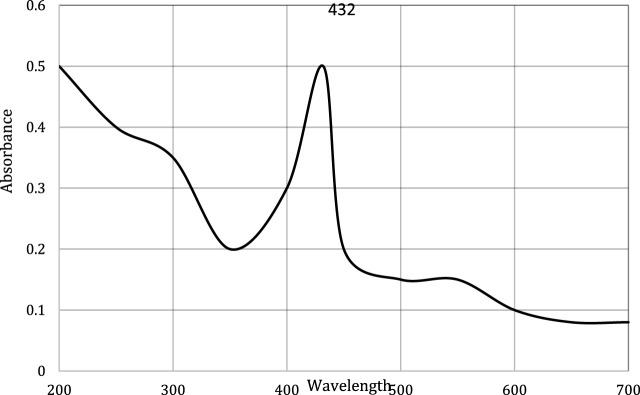
UV-visible spectra of silver nanoparticles synthesized from chicory compound.

This study carried out the characterization of silver nanoparticles synthesized from chicory compounds using this technique. During the synthesis, formation of silver nanoparticles was detected through color change from light yellow to brown. The wavelength was fixed between 200 and 700 nm. Absorption band was obtained at 432 nm (Figure 2), which corresponds to the surface plasmon resonance of AgNPs. The shape and position of the characteristic absorption band depended on the size and shape of nanoparticles.

#### Fourier transform infrared spectroscopy

**FIGURE 3:**
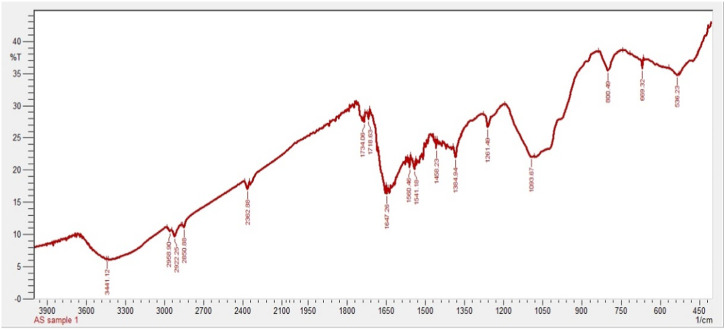
FTIR spectrum of silver nanoparticles synthesized from chicory compound.

In the present work, FTIR analysis was performed to detect the presence of functional groups involved in capping and stabilization of silver nanoparticles synthesized from chicory compounds. Figure 3 shows FTIR spectra of silver nanoparticles that exhibited a number of absorption peaks at different wavelengths: 3,441 cm^−1^, 2,958 cm^−1^, 2,922 cm^−1^, and 2,850 cm^−1^; 2,362 cm^−1^ and 1734 cm^−1^; 1718 cm^−1^, 1,647 cm^−1^, and 1,560 cm^−1^; 1,541 cm^−1^, 1,458 cm^−1^, and 1,384 cm^−1^; 1,093 cm^−1^, 800 cm^−1^, 669 cm^−1^, and 536 cm^−1^; and 1261 cm^−1^ (Figure 3).

#### EDX analysis of AgNPs

**FIGURE 4:**
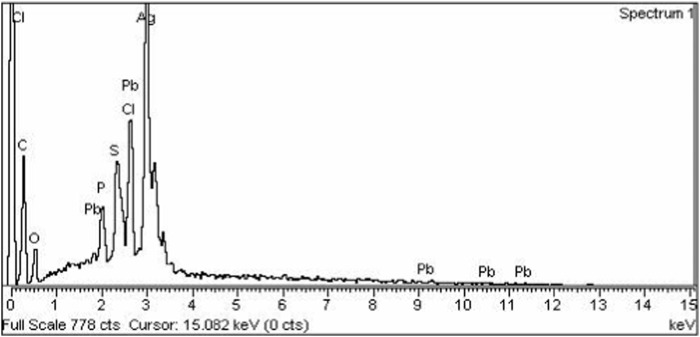
EDX spectrum of silver nanoparticles synthesized from chicory compound.

Energy dispersive X-Ray analysis (EDX) was carried out to determine the elemental composition of synthesized silver nanoparticles. An EDX spectrum of AgNPs shows a strong peak at 3 KeV that correspond to the silver element along with weak Cl, S, P, O, C, and Pb peaks, which may be due to the presence of biomolecules on the surface of nanoparticles, which are involved in the reduction of silver ion to elemental silver (Figure 4).

#### Scanning electron microscopy

**FIGURE 5:**
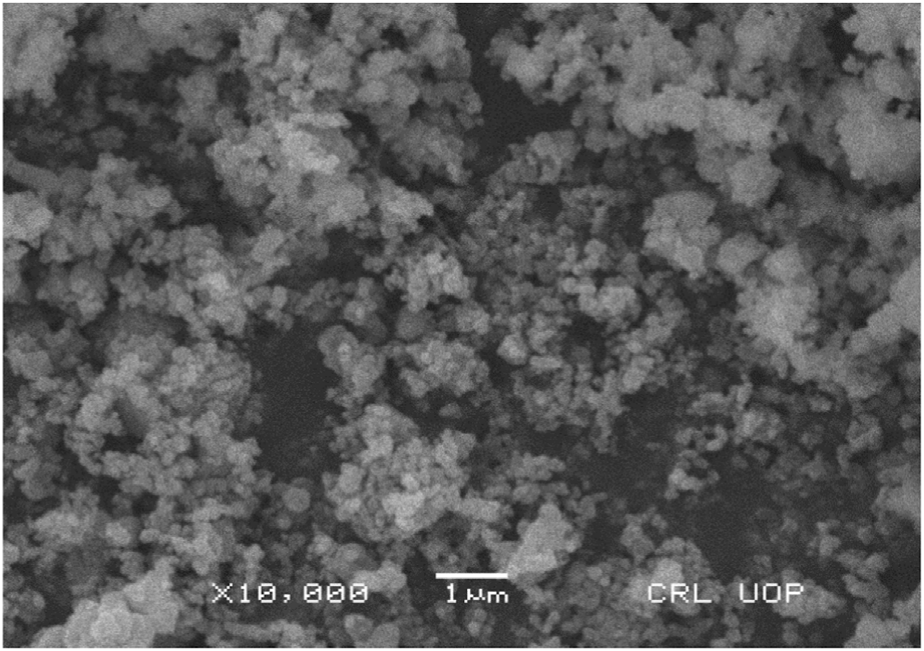
Scanning electron micrograph of AgNPs synthesized from chicory compound.

The morphology of silver nanoparticles observed by SEM analysis by the University of Peshawar showed spherical-shaped particles with an average size of about 47 ± 0.009 nm. The majority of the particles were ∼35 nm and ∼49 nm in diameter, and the minimum and maximum particle sizes observed were 25 nm and 64 nm in diameter respectively (Figure 5). Silver nanoparticles were not in direct contact with each other even in aggregated form: this shows the stabilization of AgNPs by the capping agents.

### Serum biochemical parameters of liver function

This study was undertaken to investigate, *in vivo*, the hepto-protective activity of silver nanoparticles (AgNPs) and chicory formulation extract (CCE) by using a rat model. Firstly, hepatic damage was induced by applying CCl_4_, and the damage reveals an increase in the level of different liver enzymes. Administration of CCl_4_ expresses the damaging effects in the liver in terms of an increase in serum level of ALT, ALP, cholesterol, bilirubin, and triglycerides (Table 3).

**TABLE 3:** Effect of AgNPs and aqueous extract of chicory on serum biochemical parameters of liver function in CCl_4_ intoxicated rats.

Groups	ALT (U/L)	ALP (U/L)	Total bilirubin (mg/dL)	Cholesterol (mg/dL)	Triglyceride (mg/dL)
Control	20.67 ± 1.53	140.33 ± 2.08	1.23 ± 0.21	139.67 ± 0.58	155.33 ± 3.06
CCl_4_ 1 mL/kg	111.67 ± 1.5^*^	193 ± 1.00^*^	3.27 ± 0.2^*^	193.67 ± 3.97^*^	190 ± 2.00^*^
CCl_4_+20 mg/kg Silymarin	67.33 ± 2.08^**^	162.67 ± 1.53^**^	2.50 ± 0.20^**^	156.0 ± 2.00^**^	160.0 ± 2.00^**^
CCl_4_+1.75 mg/kg AgNPs	104.67 ± 1^**^	189 ± 2.00^**^	2.90 ± 0.1^**^	188.33 ± 2.08^**^	181.33 ± 1.53^**^
CCl_4_+2.35 mg/kg AgNPs	91.67 ± 1.53^**^	180.33 ± 1.53^**^	2.70 ± 0.10^**^	174 ± 1.73^**^	175.67 ± 2.08^**^
CCl_4_+3.25 mg/kg AgNPs	74.0 ± 1.53^**^	172.67 ± 2.08^**^	2.63 ± 0.21^**^	167.67 ± 2.52^**^	169.67 ± 1.52^**^
CCl_4_+1.75 mg/kg chicory	50.75 ± 1.71^**^	175 ± 2.58^**^	2.73 ± 0.10^**^	177.0 ± 1.83^**^	181.25 ± 3.40^**^
CCl_4_+2.35 mg/kg chicory	44.50 ± 4.04^**^	167.25 ± 2.75^**^	2.65 ± 0.19^**^	166.75 ± 2.36^**^	177.5 ± 2.38^**^
CCl_4_+3.25 mg/kg chicory	39.50 ± 1.29^**^	159.25 ± 3.77^**^	2.28 ± 0.17^**^	159.50 ± 2.08^**^	159 ± 2.16^**^

The data were analyzed statistically by one-way ANOVA; *p* < 0.05 was considered significant in all cases. A post-hoc test was performed for multiple statistical comparisons between parametric data. The software Statistix 8.1 was used for the analysis of the data. All values expressed as mean ± S.D (*n* = 6). *p ≤ 0.05 Vs. control, **p ≤ 0.05 Vs. CCl_4_.

The AgNPs and chicory formulation extract possessed remarkable hepato-protective potential in a dose-dependent manner (Table 3). The AgNPs and CCE showed significant hepato-protective activity in a dose dependent manner (Table 3). An increase in the level of ALT and ALP was observed in the CCl_4_-treated group as compared to the normal group. Treatment with AgNPs at a dose of 3.25 mg/kg showed a significant reduction in levels of ALT and ALP as compared to the CCl_4_ group. On the other hand, AgNPs at the dose of 1.75 mg/kg failed to ameliorate the hepatic injury significantly.

The hepato-protective activity of AgNPs at a dose of 3.25 mg/kg was comparable with standard silymarin. As compared to the normal control group, the level of total bilirubin, cholesterol, and triglycerides was higher in CCl_4_-intoxicated rats. However, the treatment with silver nanoparticles (3.25 mg/kg) restored the normal level of cholesterol, bilirubin, and triglycerides. AgNPs at the doses of 1.75 mg/kg and 2.35 mg/kg reduced the level of these biochemical parameters but not significantly. The colloidal solution of silver nanoparticles showed maximum hepato-protective activity at a dose of 3.25 mg/kg in terms of a decrease in the level of liver enzymes (Table 3). Post treatment of rats with aqueous extracts of chicory compounds showed remarkable protection against CCl_4_-induced toxicity. Restoration of the biochemical parameters towards the normal value was observed in the group treated with 1.75 mg/kg and 2.35 mg/kg doses of chicory extract. However, the treatment of CCl_4_-intoxicated rats with chicory extract at a dose of 3.25 mg/kg was shown to be highly significant in terms of a decrease in the values of ALT and ALP.

The administration of CCl_4_ resulted in an increase in total bilirubin, cholesterol, and triglycerides, which indicated the liver dysfunction. However, treatment with a 3.25 mg/kg dose of chicory extract showed significant amelioration by reducing the level of total bilirubin, triglycerides, and cholesterol (Table 3).

#### Recovery percentage of serum and biochemical parameters of hepatotoxicity induced by CCL_4_

**FIGURE 6:**
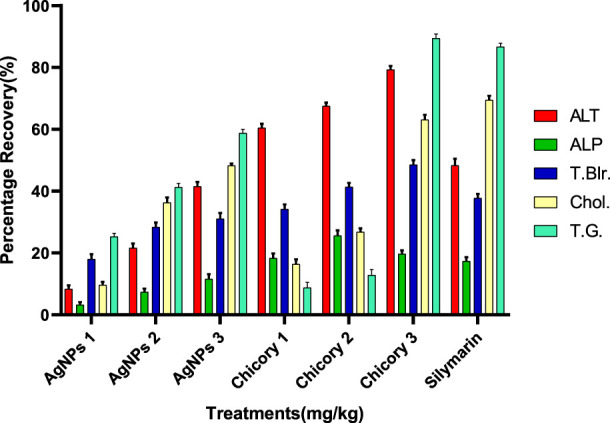
Summary of the recovery percentage of chicory formulation extract (CCE) and silver nanoparticles (AgNPs). Silymarin (standard drug), AgNPs 1 (AgNPs 1.75 mg/kg), AgNPs 2 (AgNPs 2.35 mg/kg), AgNPs 3 (AgNPs 3.25 mg/kg), Chicory 1 (1.75 mg/kg), Chicory 2 (2.35 mg/kg), Chicory 3 (3.25 mg/kg).

The recovery percentage of the induced hepatic toxicity caused by CCL_4_ was calculated through damaged and control values obtained from the blood analysis for ALT, ALP, total bilirubin, cholesterol, and total glyceride in line with the given doses. A detailed analysis indicated that the activity recorded was concentration dependent for both chicory formulation extracts (CCE) as well as silver nanoparticles (AgNPs); however, the activity of CCE was higher than the control in all the studied parameters (Figure 6).

#### Recovery percentage of chicory formulation extract (CCE)

**FIGURE 7:**
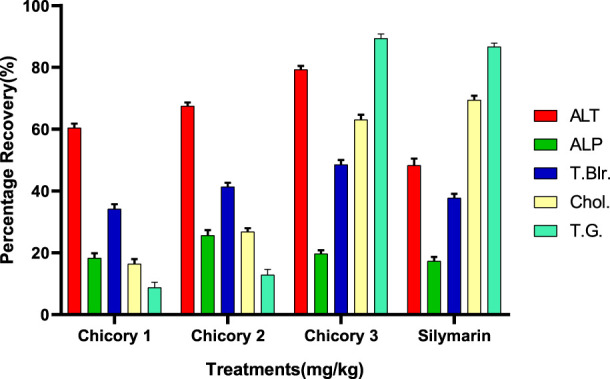
Recovery percentage of chicory formulation extract at the various concentrations. Silymarin (standard drug), Chicory 1 (1.75 mg/kg), Chicory 2 (2.35 mg/kg), Chicory 3 (3.25 mg/kg).

All the treatments showed significant recovery in a dose-dependent manner (Figure 6). Maximum recovery percentages for ALT, total bilirubin, and total glyceride recorded at a dose of 3.25 mg/kg were 79.30%, 48.93%, and 89.42%, respectively, higher than the drug (Figure 7). The optimum recovery percentage for ALP was recorded in a dose of 2.35 mg/kg higher than the standard, and for the cholesterol, a maximum recovery percent was noted at 3.25 mg/kg closer to the drug.

#### Recovery percentage of silver nanoparticles (AGNPs)

**FIGURE 8:**
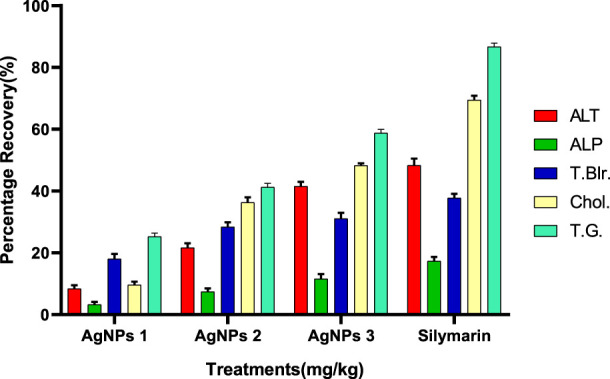
Recovery percentage of silver nanoparticles (AgNPs) at the various concentrations. Silymarin (standard drug), AgNPs 1 (AgNPs 1.75 mg/kg), AgNPs 2 (AgNPs 2.35 mg/kg), AgNPs 3 (AgNPs 3.25 mg/kg).

Compared to the hepato-protective drug (silymarin), the recovery percentile of AgNPs for each parameter was found in a dose-dependent manner (Figure 8). For ALT, the maximum recovery percentage was recorded at the dose of 3.25 mg/kg (41.39%), which was comparable to the standard (48.72%). The same trend was observed for ALP, total bilirubin, cholesterol, and total glyceride, with optimum levels at the highest dose closer to the drug (Figure 8).

### Histopathological examination

**FIGURE 9:**
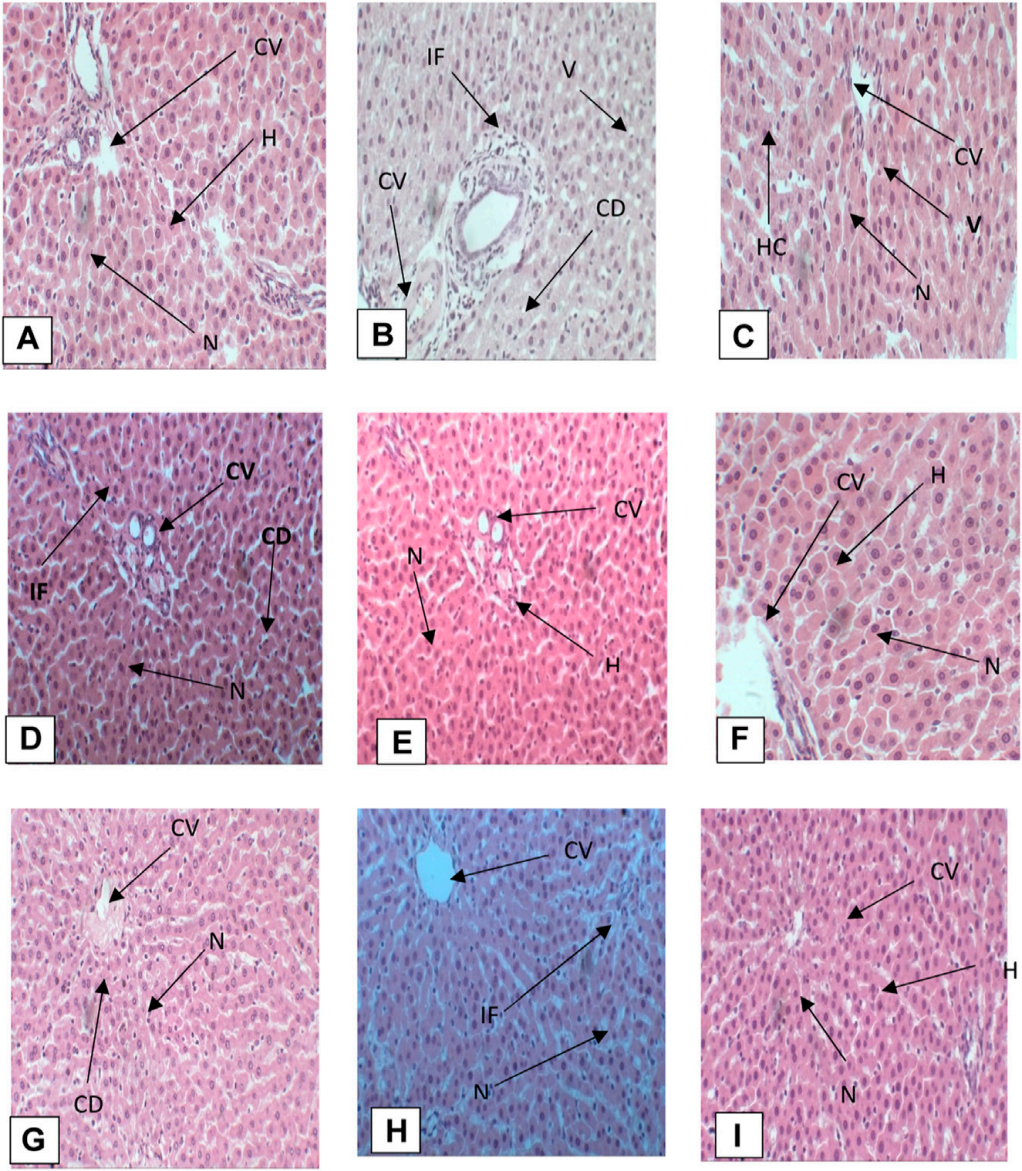
**(A)** Photomicrograph of liver section of control group, **(B)** CCl_4_ group, **(C)** silymarin group, **(D)** CCl_4_ + chicory (1.75 mg/kg) group, **(E)** CCl_4_ + chicory (2.35 mg/kg) group, **(F)** CCl_4_ + chicory (3.25 mg/kg), **(G)** group CCl_4_ + AgNPs (1.75 mg/kg) group, **(H)** CCl_4_ + AgNPs (2.35 mg/kg) group, **(I)** CCl_4_ + AgNPs (3.25 mg/kg) group. Abbreviations: (N) nucleus, (S) sinusoid, (V) vacuolization, (H) hepatocyte, (BD) bile duct, (IF) infiltration, (CV) central vein (McDonald et al., 2020), cytoplasmic degeneration (CD), (PT) portal triad.

Histopathological studies were carried out to examine the degree of damage caused by oral administration of CCl_4_ and recovery through administration of different doses of AgNPs and chicory extract. Microscopic analysis of liver tissue taken from each group was done, histopathology of CCl_4_-intoxicated rats was compared with control, and comparison of remaining treatments was done with the CCl_4_ group. The hepato-protective activity of chicory extract and AgNPs was confirmed by histopathological examination (Figure 9).

#### Histopathology of control group

Histopathology of the control group showed normal architecture of hepatocytes when compared with the CCl_4_ group. There was a normal arrangement of hepatocytes, with intact structure, devoid of degenerative changes, well preserved cytoplasm, and prominent nucleus. There was a distinct central vein (Figure 9A).

#### CCl_4_ group

Histopathology of the CCl_4_ group showed vacuolization, nuclear degeneration, infiltration, distal sinusoid, loss of cellular boundaries, necrosis, ballooning degeneration, deformation of central vein, and disruption of normal hepatic cords. Kupffer cells at various points were also observed (Figure 9B).

#### CCl_4_ + silymarin 20 mg/kg

Histopathological analysis of liver sections of rats treated with silymarin showed recovery of hepatocytes with restoration of normal architecture. There was reduced vacuolization, normal central vein, prominent nucleus, absence of necrosis, and reduced sinusoid (Figure 9C).

#### CCl_4_ + chicory extract (1.75 mg/kg)

Histopathological analysis of photomicrograph of rats treated with chicory extract at the dose of 1.75 mg/kg showed mild recovery of hepatocytes toward normal architecture. There was less vacuolization and a prominent nucleus, but at some places cytoplasmic degeneration was also observed (Figure 9D).

#### CCl_4_ + chicory (2.35 mg/kg)

Histopathology of rats treated with CCl_4_ + chicory extract (2.35 mg/kg) showed remarkable recovery. A prominent nucleus, intact central vein, reduced vacuolization, well preserved cytoplasm, infiltration at few places, and a less dilated sinusoid were observed. Hepatic architecture was just near to normal (Figure 9E).

#### CCl_4_ + chicory extract (3.25 mg/kg)

Histopathology of live section of rats treated with chicory extract at the dose of 3.25 mg/kg of body weight revealed a marked decrease in inflammation, normal arrangement of hepatocytes, intact hepatic cord, mild inflammation, prominent nucleus, intact central vein, and well preserved cytoplasm (Figure 9F).

#### CCl_4_ + AgNPs (1.75 mg/kg)

Histopathological analysis of photomicrograph of rats treated with AgNPs at the dose of 1.75 mg/kg showed mild recovery of hepatocytes toward normal architecture. Less vacuolization and prominent nucleus were observed, but at some places cytoplasmic degeneration was also observed (Figure 9G).

#### CCl_4_ + AgNPs (2.35 mg/kg)

Histopathology of rats treated with CCl_4_ + AgNPs (2.35 mg/kg) showed remarkable recovery. Prominent nucleus, intact central vein, reduced vacuolization, well preserved cytoplasm, infiltration at few places, and less dilated sinusoid were observed. Hepatic architecture was just near to normal (Figure 9H).

#### CCl_4_ + AgNPs (3.25 mg/kg)

Histopathology of live section of rats treated with AgNPs at the dose of 3.25 mg/kg revealed a marked decrease in inflammation, mild degeneration, prominent nucleus, intact central vein, and well preserved cytoplasm (Figure 9I).

The histopathological investigation supported the recovery percentage by the application of chicory formulation extract and silver nanoparticles (Figure 9). The chicory formulation has traditionally been used to treat various liver disorders for centuries, and this study revealed that the chicory formulation extract exhibited a remarkable recovery percentage of liver in the liver architecture that was higher than the drug (i.e., silymarin).

## Discussion

The current research work was carried out to evaluate the hepato-protective activity of AgNPs and chicory formulation extract against CCl_4_-induced hepatic damage in a rat model. This study involved the synthesis of AgNPs from a chicory formulation and characterization through UV-visible, SEM, EDX, and FTIR analysis. In the UV-visible spectrum, AgNPs showed an absorption peak at 432 nm that corresponds to the surface plasmon resonance of AgNPs (Figure 2). The EDX spectrum of AgNPs shows a strong peak at 3 KeV that corresponds to silver elements along with weak Cl, S, P, O, C, and Pb peaks. In SEM analysis, the majority of the particles were of size ⁓35 nm and ⁓49 nm in diameter, and the minimum and maximum particle size observed was 25 nm and 64 nm in diameter, respectively (Figure 5).

The FTIR spectrum showed absorption peaks at different wavelengths. For instance, a 3,441 cm^−1^ peak indicates O-H extending of alcohol and phenols. The absorption peaks at 2,958 cm^−1^, 2,922 cm^−1^, and 2,850 cm^−1^ correspond to alkanes C-H elongating and symmetrical stretching of the methylene group (Syafiuddin et al., 2017). Absorption peaks at 2,362 cm^−1^ and 1734 cm^−1^ were assigned to N-H and C=O, respectively. The absorption bands at 1718 cm^−1^, 1,647 cm^−1^, and 1,560 cm^−1^ indicate the presence of C=O, aromatic C=C, and diketones, respectively (Ali et al., 2015). Some of the peaks at 1,541 cm^−1^, 1,458 cm^−1^, and 1,384 cm^−1^ represent alkanes and alkyl groups, C=C stretching of aromatic compounds, and C-O, respectively. Bands at 1,093 cm^−1^, 800 cm^−1^, 669 cm^−1^, and 536 cm^−1^ indicate the C-O, N-H wagging of amines, C-H bending, and aromatic compounds (Zulfiqar et al., 2019) (Figure 3). The peak observed at 1261 cm^−1^ denotes the presence of C-H waging of alkyl halides.

The administration of CCl_4_ expresses the damaging effects in the liver in terms of an increase in serum level, ALT, ALP, cholesterol, bilirubin, and triglycerides. CCl_4_ reacts with cytochrome P450 and produces trichloromethyl radical that converts into proxy radical in the presence of oxygen. These free radicals attach to macromolecules and initiate lipid peroxidation that causes cellular damage and membrane leakage. It results in an increased level of liver marker enzymes such as ALP and ALT. The level of biochemical markers such as cholesterol, bilirubin, and triglycerides (Chen et al., 2012; Syed et al., 2014). The results of current study demonstrated that AgNPs and chicory formulation extract possessed remarkable hepato-protective potential (Table 3). The hepato-protective activity of a plant depends upon the ability to ameliorate the toxic effect or maintain normal physiological functions (Ouassou et al., 2021).

The present study revealed that treatment with chicory formulation extract and AgNPs restored the normal functioning of the liver and repaired cellular damage, evidenced by a decrease in the level of liver biochemical markers towards the normal values. However, the restorative activity of chicory formulation extract and AgNPs was observed in dose dependent manner (Table 3). These findings are in line with other studies in which AgNPs were used to ameliorate the oxidative damage caused by CCl_4_ administration (Zhang et al., 2019).

A colloidal solution of silver nanoparticles showed maximum hepato-protective activity at the dose of 3.25 mg/kg in term of a decrease in the level of liver enzymes (Table 3). These results correlate with studies where treatment with nanoparticles was found to be more effective in restoring the level of different liver enzymes in a dose dependent manner. However, more hepato-protective activity was observed at the medium and highest doses (Kumar et al., 2011). Furthermore, AgNPs at the dose of 1.75 mg/kg failed to ameliorate the hepatic injury significantly. The results of this study are correspond to previous studies carried out on silver nanoparticles by using a leaf extract of *Rhizophora apiculata*. Silver nanoparticles were found more effective in ameliorating hepatic injury at the highest dose (Zhang et al., 2019). Increased levels of ALT, ALP, bilirubin, cholesterol, and triglycerides in the serum suggest a compromised integrity of hepatocellular membranes and cellular leakage. The use of AgNPs showed promise in restoring these enzyme levels to normal, indicating their potential to protect organs from damage. The stabilization of ALT, ALP, bilirubin, cholesterol, and triglycerides through AgNP treatment signified an enhancement in the functional maintenance of hepatocytes. Notably, AgNP treatment led to a reduction in bilirubin levels, moving them closer to the normal range.

The recovery percentage of the induced hepatic toxicity caused by CCL_4_ was calculated through damaged and control values obtained from the blood analysis for ALT, ALP, total bilirubin, cholesterol, and total glyceride in line with the given doses. The detailed analysis indicated that the activity recorded was concentration dependent for both CCE as well as AgNP; however, the activity of CCE was higher than the control in all the studied parameters due to the presence of more hepatoprotective agents (Figure 6). Maximum recovery percentages for ALT, total bilirubin, and total glyceride recorded at the dose of 3.25 mg/kg were 79.30%, 48.93%, and 89.42%, respectively, higher than the drug (Figure 7). The compounds present in CCE were actually contributed by four different plants, and they exerted their therapeutic potential through their free radicle scavenging activity. The optimum recovery percentage for ALP was recorded at the dose of 2.35 mg/kg higher than the standard, and for the cholesterol, the maximum recovery percentage was noted at 3.25 mg/kg closer to the drug (Figure 7). Detailed analysis of these recovery percentages showed that chicory formulation possesses more hepato-protective activity and was even higher than standard drug. This might be due to the herbal ingredients of chicory formulation, which possess an active constitute responsible for its hepato-protective activity. These results are also supported by another study in which a polyherbal formulation (Aab-e-Murawaqain) showed a significant hepato-protective effect (Amir et al., 2022).

*Cichorium intybus* is a major component of chicory compound. The hepato-protective activity of its various parts has been reported. The ethanolic extract of roots is reported to contain sesquiterpene lactones, insulin, chicoric acid, pectin, choline, and reducing sugar (Mathur and Mathur, 2016). The hepato-protective activity of methanolic fractions and phenolic compound of seeds of *C. intybus* has been reported in albino rats of the Wistar strain after the induction of hepato-toxicity by CCl_4_ administration. Biochemical parameters such as γ-glutamyl transferase (GGT), alanine aminotransferase (ALT), aspartate aminotransferase (AST), and alkaline phosphatase (ALP) were analyzed to evaluate hepato-protective action (Reitman and Frankel, 1957; Elgengaihi et al., 2016).

Previous studies showed the hepato-protective activity of the essential oil of *F. vulgare*. Its seeds are reported to possess trans-anethole, fenchone, methylchavicol, limonene, camphene, ß-pinene, ß-myrcene, phellandrene, 3-carene, camphor, and cisanethole (Simándi et al., 1999; Heghes et al., 2019). Among these, ß-myrcene and limonene have been shown to affect liver functions in CCl_4_-induced albino rats, determined from serum biochemical analysis of ALT, AST, ALP, and bilirubin concentration (Reicks and Crankshaw, 1993; Joshi and Soulimani, 2020).

*Cucumis melo* has been reported to possess hepato-protective activity in CCl_4_-intoxicated rats, where it increases gluconeogenesis (Milind and Kulwant, 2011). Different phytochemicals from the fresh fruit of *Cucumis sativus* were reported to possess hepato-protective potential against cumene hydroperoxide (CHP)-induced oxidative stress. The outcomes of this experiment showed that aqueous extract of *C. sativus* L. acts as a hepatoprotective and antioxidant agent against induced hepatotoxicity, which suggests that antioxidants and radical scavenging components of *C. sativus* L. fruit extract can easily cross the cell membrane and cope with the intracellular ROS formation (Heidari et al., 2012; Gopalakrishnan et al., 2016). Various previous studies reported that ingredients of chicory formulation individually possess hepato-protective activity. Therefore, the combined synergetic effect of all ingredients may have profound effect in normalizing liver functions.

The administration of CCl_4_ led to the production of reactive free radicals that bind with macromolecules and induce lipid peroxidation, and result in the disruption of normal liver architecture (Singh et al., 2014). The livers of normal control animals displayed a distinct central vein, well-preserved cytoplasm, and noticeable nucleus and nucleolus (Figure 9). In contrast, liver sections from animals treated with CCl_4_ exhibited severely toxic cells, including necrotic liver cells, inflammatory cell accumulation, and widespread inflammation throughout the liver parenchyma, with infiltration and necrosis. Having hepatocytes with a preserved cytoplasm suggests that chicory extracts were more effective than AgNPs in mitigating CCl_4_ toxicity, and silymarin also demonstrated protective effects against CCl_4_-induced hepatic alterations. The chicory formulation has been traditionally used to treat various liver disorders for centuries, and this study has revealed that the chicory formulation extract exhibited the remarkable recovery percentage of liver in the liver architecture, which was higher than the drug (i.e., silymarin). Nevertheless, the absence of necrosis and the presence of normal hepatic cells in rats treated with chicory extract across various groups indicated significant signals of protection. The histopathological investigation supported the recovery percentage by the application of chicory formulation extract and silver nanoparticles (Figure 9), while treatment with AgNPs also repaired the degenerative changes and restored the form of the liver. Rats treated with high doses of AgNPs showed histology near to the control group. All these results are in agreement with a previous study carried out by Reshi et al. (2017).

This study successfully synthesized and characterized silver nanoparticles from chicory formulation extract. Both the chicory formulation extract and AgNPs were tested for hepato-protective effective in a rat model with CCl_4_-induced damage. The recovery of CCl_4_-induced liver damage through the dose dependent application of chicory formulation extract as well as AgNPs signifies the hepato-protective effects. The findings of this study reveal that chicory formulation extract possessed more hepato-protective potential as compared to AgNPs by regulating the biochemical and histo-pathological parameters. This study also validated the traditional use of chicory formulation in traditional systems of medicine to treat liver disorders. However, there is a need for further studies with molecular characterization to find the lead compound with hepato-protective effects from both chicory extract and AgNPs. In addition, there is need to explore the mechanism of action of the compound responsible for this activity.

## Study limitations

The findings of the study may be specific to the chosen experimental conditions, and the transferability of the results to broader populations or different settings may be limited.

The study may not have explored a wide range of doses or exposure durations.

## Data Availability

The original contributions presented in the study are included in the article/Supplementary material, further inquiries can be directed to the corresponding authors.

## Abbreviations

AgNPs, Silver nanoparticles; ALT, Alanine transaminase; Chol, Cholesterol; T.G, Triglycerides; FTIR, Fourier transform infrared spectroscopy; ALP, *Alkaline phosphatase*; CCl_4_, Carbon tetrachloride; Blr, Bilirubin; LFTs, Liver function tests.
